# Hypoxia-induced inflammation and purinergic signaling in cross clamping the human aorta

**DOI:** 10.1186/s40064-015-1651-x

**Published:** 2016-01-04

**Authors:** Juho Jalkanen, Mikael Maksimow, Sirpa Jalkanen, Harri Hakovirta

**Affiliations:** Department of Vascular Surgery, Turku University and Turku University Hospital, Hämeenkatu 11, 20521 Turku, Finland; MediCity Research Laboratory, Department of Microbiology and Immunology, University of Turku, Tykistönkatu 6A, 20520 Turku, Finland

**Keywords:** Hypoxia, Aortic clamping, SIRS, IL-6, HGF, HIF-1a, CD73

## Abstract

Open aortic surgery evokes a systemic inflammatory response and is associated with high morbidity and mortality. Purinergic signaling has been shown to be crucial for maintaining vascular integrity and attenuating inflammation related to hypoxia. The involvement of purinergic signaling in cross clamping of major human arteries is unknown. Our aim was to compare systemic inflammatory responses and hypoxia-induced purinergic signaling in patients undergoing either open infra-renal abdominal aortic repair or infra-inguinal revascularization. Pre- and 24 h post-operative blood samples were gathered from 6 patients undergoing aortic clamping and 6 similar patients undergoing common femoral artery cross-clamping. Using Biorad Multipex™ 21- and 27-panels 48 different cytokines, chemokines and growth factors were analyzed, in addition to circulating levels of ATP, ADP, CD39, CD73 and HIF-1α, and compared between the groups. Several inflammatory cytokines were elevated from baseline levels after aortic clamping, but not after femoral cross clamping. Most pronoun rises were seen in IL-6 (667 %, *P* = 0.016) and HGF (760 %, *P* = 0.016). HIF-1α values showed a steady increase after clamping of either artery unless the subject underwent blood transfusion. Despite an adequate increase in HIF-1α CD39 and CD73 activity decreased significantly after aortic clamping (P = 0.047 and P = 0.016, respectively). Aortic clamping is associated with a clear and strong systemic inflammatory response and impaired repair mechanisms in terms of purinergic signaling. Patients undergoing open aorta repair could benefit from pre-operative medical therapy, which enhances CD73 expression.

**Electronic supplementary material:**

The online version of this article (doi:10.1186/s40064-015-1651-x) contains supplementary material, which is available to authorized users.

## Background

Acute organ injury remains one of the leading causes of morbidity and mortality in surgical patients (Eltzschig 2013). A highlight of such surgery is open aortic cross clamping and reconstruction, which is associated with a systemic inflammatory response syndrome (SIRS) (Bown et al. 2001; Vasdekis et al. 2008) and dysfunction of central organs, especially the lungs, kidneys and intestine (Brady et al. 2000; Papia et al. 2006; Moris et al. 2014). Thus, open aorta repair has been referred to as the ultimate stress test (Ali et al. 2008). Patients undergoing major vascular surgery are also often burdened by other significant cardiovascular illnesses. This is especially the case amongst patients with peripheral artery disease (PAD), for whom the prevalence of cardiovascular co-morbidities is significantly elevated (Gallino et al. 2014). It has been shown that patients undergoing surgery of the lower limb for PAD have even more peri/post-operative myocardial morbidity than do patients undergoing aorta repair due to abdominal aortic aneurysm (AAA), but yet patients undergoing aortic surgery have a worse scenario (Krupski et al. 1992; Ali et al. 2008). This has lead to the conception that cross clamping of the infra-renal abdominal aorta leads to a markedly different hypoxia-induced pathophysiologic response than the cross-clamping of the iliac or common femoral artery.

Extra cellular CD73 (ecto-5′-nucleotidase/NT5E) derived adenosine production is considered a key pathway in attenuating hypoxia-induced inflammation (Thompson et al. 2004; Eltzschig et al. 2012) and protective of several central organs (Grenz et al. 2007; Eckle et al. 2007; Hart et al. 2011). The key driver of this response is hypoxia-induced factor-1 alpha (HIF-1α). However, the current background literature of this pathway is mostly derived from animal studies, in which cross-clamping of central arteries has been widely used to provoke reperfusion injury and a systemic inflammatory response in order to study different organ failures (Grenz et al. 2007; Kiss et al. 2007; Hart et al. 2011).

Thus, in this study our objective was to study the systemic inflammatory and growth factor response of aortic cross clamping. In addition, to further understand hypoxia-induced repair mechanisms in relation to cross clamping of a major artery, components of purinergic signaling were analyzed. Pre- and post-operative levels of 48 different cytokines, chemokines and growth-factors, in addition to ATP, ADP, CD39, CD73 and HIF-1α, were analyzed from blood samples of six PAD patients undergoing open repair of the infra-renal abdominal aorta and were compared to six similar risk factor matched PAD patients undergoing infra-inguinal vascular surgery and cross-clamping of the common femoral artery.

## Results and discussion

### Description of the study subjects

The study group baseline data is presented in Table 1. The groups were well matched and were mostly without any statistically significant differences. Patients undergoing open aorta repair tended to be a bit younger and had slightly less renal insufficiency and coronary artery disease (CAD), see Table 1A. The groups were also comparable based on major issues relating to procedural variables such clamping time and blood loss (Table 1B). The only significant difference was seen in the amount of transfusion of saline products received between the sampling. Subjects undergoing open aorta repair received significantly more crystalloid transfusion than did subjects undergoing infra-inguinal surgery (*P* = 0.03).

**Table 1:** Description of baseline characteristics between patients undergoing clamping of the aorta vs. common femoral artery

	Aorta (n = 6)	Femoral (n = 6)	*P* value
A (Baseline characteristics)			
Gender (F/M %)	50/50 %	33/67 %	NS*
Age (years)	64.5 (61–71)	72.5 (64–77)	NS**
Hypertension	67 %	67 %	NS*
Coronary artery disease	33 %	50 %	NS*
Dyslipidemia	17 %	17 %	NS*
COPD	33 %	33 %	NS*
Diabetes	17 %	17 %	NS*
Renal insufficiency	17 %	33 %	NS*
Systolic BP (mmHg)	160 (146–174)	148 (133–152)	NS**
Creatine (μmol/L)	78 (61–101)	92 (68–136)	NS**
CRP (mg/L)	8 (3–19)	8 (3–18)	NS**
B (Procedure related variables)			
Procedure time (min)	213 (148–233)	150 (103–218)	NS**
Clamping time (min)	75 (45–98)	78 (38–135)	NS**
Blood loss (mL)	1025 (500–2890)	250 (88–1425)	NS**
Amount of saline transfusion between sampling (mL)	6250 (4575–8800)	2975 (2575–5100)	0.03**
Time from clamping to second sample (h)	29 (22–38)	21 (20–28)	NS**

Prevalence presented as percentage and numerical values as median and inter-quartile range (IQR). Difference between groups calculated using the * Chi square test and ** Mann–Whitney U test

### Aortic clamping significantly elevated levels of several cytokines, especially IL-6 and HGF

The majority of the values for IFN-α2, LIF, IL-1α, IL-3, IL-12p40, IL-15, bFGF, RANTES and TNF-β were below the detection limit, and were left out of the statistical analysis.

**Fig. 1:**
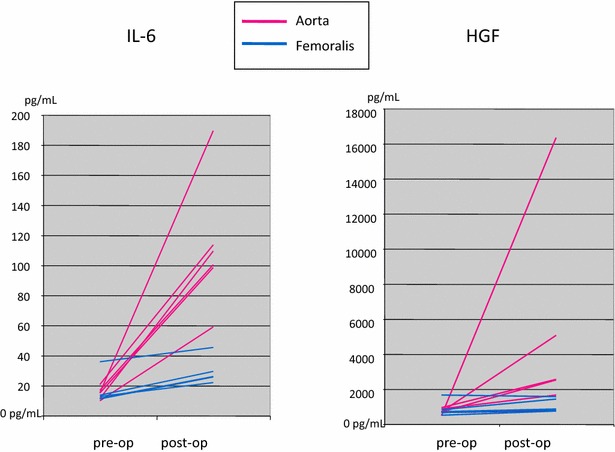
Change in IL-6 and HGF levels (pg/mL) after cross clamping of the aorta (*red*) and common femoral artery (*blue*) presented as matched pairs of pre- and post-operative samples

 For aortic clamping there was a significant rise in the level of IL-6 (667 %, *P* = 0.016), IL-8 (75 %, *P* = 0.047), IL-2Rα (152 %, *P* = 0.031), IL-16 (129 %, *P* = 0.031), GROα (252 %, *P* = 0.031), HGF (760 %, *P* = 0.016), M-CSF (853 %, *P* = 0.016), and SDF-1α (447 %, *P* = 0.016) using matched pair values across time and the Wilcoxon signed rank test. For cross clamping of the common femoral artery there was only a significant increase in the level of IL-6 (96 %, *P* = 0.031) and GROα (84 %, *P* = 0.031). All of the cytokines, which underwent a significant alteration due to clamping were then tested for the effect of aortic clamping versus femoral artery clamping between the groups. These results are presented in Table 2. First, the pre-operative baseline values between the groups were tested to ensure that the effect of clamping was not affected by any underlying baseline differences. No significant differences in the cytokine levels between the groups could be seen in pre-operative sampling. For aortic clamping the most pronoun change was observed in the elevation of IL-6, when compared to femoral clamping (667 vs. 96 %, respectively, *P* = 0.006). As was also seen for HGF (760 % increase for aortic clamping vs. 33 % increase for femoral clamping, *P* = 0.045). Figure 1 illustrates the differences of aortic vs. femoral clamping on IL-6 and HGF as matched pairs. All the corresponding raw values are presented in Additional file 1: Table S1. Other cytokines failed to show statistical increase in the aortic clamping when compared to the femoral clamping as matched pairs.

**Table 2:** Baseline values of significantly altered cytokines presented as median and inter-quartile range (IQR) and average percentage of the change after cross clamping of the aorta vs. common femoral artery

	Aorta	Femoral	*P* value*
IL-6			
Baseline (pg/mL)	15.3 (10.8–17.7)	12.5 (11.1–24.5)	NS
% change	667 %	96 %	0.006
IL-8	40.0 (33.2–51.3)	29.5 (27.0–39.1)	NS
	75 %	17 %	0.361
IL-2Rα	89.7 (77.4–160)	65 (39.6–174)	NS
	152 %	7 %	0.068
IL-16	142 (81–172)	154 (46–194)	NS
	129 %	20 %	0.068
GROα	30.9 (20.8–52.5)	58.0 (34.7–77.9)	NS
	252 %	84 %	0.144
HGF	681 (599–865)	688 (560–1225)	NS
	760 %	33 %	0.045
M-CSF	10.3 (5.45–15.9)	11.1 (4.33–57.5)	NS
	853 %	197 %	0.055
SDF-1α	69.6 (32.0–130)	75.4 (41.3–77)	NS
	447 %	10 %	0.154

* Mann–Whitney U test

### CD39 and CD73 decrease significantly after clamping aortic clamping

**Fig. 2:**
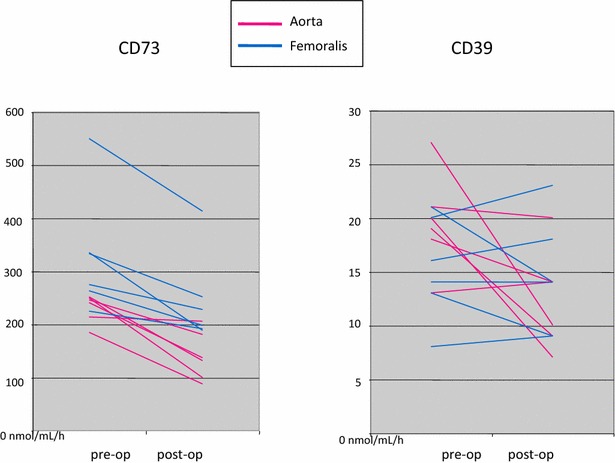
Change in CD73 and CD39 activity activities (nmol/mL/h) after cross clamping of the aorta (*red*) and common femoral artery (*blue*) presented as matched pairs of pre- and post-operative samples

Baseline values of CD39 and CD73 did not differ between groups (Table 3). After aortic clamping both CD39 and CD73 significantly declined from baseline levels, see Fig. 2 and Table 3. CD39 declined an average of 43 % (P = 0.047), ranging from 2 to 63 % decline. A similar 46 % decline was seen in CD73 (P = 0.016), ranging from 21 to 55 % decline (Fig. 2). In cross clamping of the common femoral artery there was no significant decrease in CD39, but a significant decrease in CD73 activity (Fig. 2). CD39 decreased an average of 6 %, ranging from 13 % increase to 24 % decrease. After common femoral artery clamping CD73 had a more constant, yet significant average 25 % decrease, ranging from 16 to 30 % decrease, P = 0.016). Thus, a stronger decline was seen in aortic clamping than in clamping of the femoral artery in both CD39 (43 vs 6 % decrease, respectively, P = 0.037) and CD73 (46 vs. 25 % decrease, respectively, P = 0.11), but for CD39 this was more evident. Circulating ATP and ADP levels showed no consistent or significant changes between pre- and post-operative samples in either procedure. Both increases and decreases were seen between pre- and post-operative levels, which amounted to slight but non-significant decrease in both ATP and ADP (Table 3). All raw values of components of purinergic signaling are presented in Additional file 1: Table S1.

**Table 3:** Baseline values of ATP, ADP, CD39 and CD73 presented as median and range, and average percentage change (and range) after clamping

	Aorta baseline	Average change	*P* value*	Femoral baseline	Average change	*P* value*
ATP (nmol/L)	3276 (1582–4476)	−6 % (−30 to 50 %)	NS	4336 (2731–5187)	−21 % (−42 to 21 %)	NS
ADP (nmol/L)	2790 (1243–3317)	−9 % (−45 to 29 %)	NS	1755 (1219–3944)	−4 % (−56 to 71 %)	NS
CD39 (nmol/mL/h)	20.5 (13–27)	−43 % (−2 to −63 %)	0.047	15 (8–20)	−6 % (−24 to 13 %)	NS
CD73 (nmol/mL/h)	243 (184–251)	−46 % (−21 to −55 %)	0.016	303 (224–549)	−25 % (−16 to −30 %)	0.016

Significance of change after clamping calculated using matched pairs

* Wilcoxon sign rank test

### HIF-1a increased after clamping unless the subject underwent blood transfusion

**Fig. 3:**
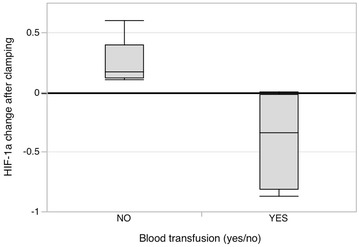
Percent change of HIF-1a levels in all patients after clamping depending on was whether a blood transfusion was received (n 4) or not (n 8). Difference between the presented groups P = 0.007 using the Mann–Whitney U test

HIF-1α was mostly associated with an increase after clamping in both groups. However, significant blood loss and consequent blood transfusion was associated with an opposing effect. All subjects who received blood transfusion had a decrease in HIF-1α (average decrease 34 %, range 0–87 % decrease). Subjects undergoing aortic clamping and not receiving blood transfusion had average 17 % increase in HIF-1α ranging from 15–61 %, and subject undergoing clamping of the femoral artery and not receiving blood transfusion had an average HIF-1α increase of 18 %, ranging from 11–36 %. Although there was a tendency of higher HIF-1α response after aortic clamping there was no significant difference between HIF-1α in the groups. The HIF-1α response between the pre- and post-operative values was however significantly affected depending on whether the blood transfusion was received or not (P = 0.007), see Fig. 3.

### Discussion of results

This study clearly shows that open infra-renal aorta repair significantly elevates levels of several circulating cytokines and growth factors, but in particular IL-6 and HGF. Both markers have been associated with worse disease prognosis in SIRS, sepsis, ARDS and severe acute pancreatitis (Parsons et al. 2005; Nieminen et al. 2014).

The role of IL-6 as an acute phase pro-inflammatory mediator is unquestionable. However, the elevation of HGF relating to SIRS after open aorta repair is a novel finding. The role of HGF is controversial according to the background literature. HGF is considered anti-inflammatory by nature, and a necessary mediator of repair mechanisms (Shimizu et al. 2012). In several animal models it has been considered protective and even a possible target for drug development to prevent organ failure (Gong 2008). HGF has been shown to down regulate inflammation and leukocyte extravasation by attenuating TNFα induced expression of intercellular adhesion molecule-1 (ICAM-1), vascular adhesion molecule-1 (VCAM-1) (Mine et al. 1998), and E-selectin (Gong et al. 2006). However, this study indicates that the inflammatory response of open aorta repair is not TNFα mediated, as TNFα was not affected from cross clamping of the aorta. Thus, the HGF response in open aorta repair could be an acute repair mechanism in response to severe hypoxia. Despite of its known anti-inflammatory mechanisms, HGF has several pleiotropic actions, and has been shown to be a potent mediator of neutrophil adhesion via integrin activation (Mine et al. 1998) and is highly expressed in polymorphonuclear leukocytes at acute sites of inflammation (Matsushima et al. 2004). Neutrophil infiltration highlights sites of acute inflammation, especially in response to ischemia and reperfusion. This may explain why HGF is highly elevated in severe acute conditions and may be a predictor of poor prognosis. According to the results of this study HGF had a strong positive correlation with IL-6 (R = 0.52, P = 0.01, data not shown).

All this translates to the conclusion that it is namely the hypoxia induced systemic acute inflammatory response that makes aortic clamping the strain it is. One could argue that the elevation in circulating cytokines seen here is a response to the overall surgical trauma patients undergo in open repair, which is extensive. Most likely surgical trauma does contribute to some of the elevation, e.g. in more slowly acting cytokines (IL-8 and IL-16) and growth factors (GROα and SDF-1α) as seen here, similar to the rise of CRP seen later on after a major surgery. However, here we have shown a dramatic acute pro-inflammatory response in the elevation of IL-6 and HGF, which are known to be induced by hypoxia and central tissue resections, but not by surgical trauma per se (Yamada et al. 1998). The known excessive need for transfusion of crystalloid fluids relating to open aorta repair, as seen in this study (Table 1), would more likely have a diluting rather than a lifting affect on cytokine levels.

The excessive need for fluid transfusion to maintain an optimum hemodynamic level relating to clamping and un-clamping of the aorta can in part be a result of impaired adenosine production as seen here in decreased CD39 and CD73 activity especially in subjects undergoing aortic clamping. To some extent CD39 activity even increased in subjects undergoing femoral clamping (Fig. 2). HIF-1α activated adenosine production by CD73 is considered the key of maintaining vascular integrity and preventing vascular leak (Thompson et al. 2004; Hart et al. 2011). For patients undergoing open aorta repair the essence of this mechanism is even more crucial due to the high volume of transfused fluids. But why do CD39 and CD73 levels significantly decline in subjects undergoing aortic clamping at the first post-operative day, when they should go up? Hypoxia is a known key driver of CD39 and CD73 activity (Semenza 2010; Schwartz et al. 2011). An adequate elevation is seen in HIF-1α, unless blood loss was significant and the subject underwent blood transfusion (Fig. 3). It is a novel finding that blood transfusion impairs the HIF-1α response, but helps to explain why blood transfusion is significantly associated with worse outcome and ischemia related morbidity (Murphy et al. 2007; Marik and Corwin 2008; Patel et al. 2014). This, however, is inadequate to explain an impaired CD39/CD73 response since this was seen in all subjects, even in subjects without blood transfusion and with an adequate elevation of HIF-1α. Perhaps CD39 and CD73 were consumed to compensate for elevated ATP and ADP levels (Yegutkin 2015) as ATP and ADP levels showed no consistent or significant alterations in either direction between the pre- and post-operative samples. Or perhaps, despite an increased expression at the endothelium, CD39 and CD73 do not shed into circulation as anticipated. Thus, an increase is not yet seen in soluble values measured here at the first post-operative day. A similar late rise in soluble activity of CD73 is seen when its expression is induced with the administration of intra venous interferon beta in humans (Bellingan et al. 2014).

### Limitations of the study

The current study has marked limitations. First, and most importantly, this study is highly limited by the low number of study subjects. Due to the low number of subjects it is virtually impossible to find statistical differences between the baseline characteristics of the study groups. Despite this dilemma, the differences between the observed inflammatory responses and repair mechanisms, on the other hand, are statistically strong and quite clear. Thus, we postulate that the observed molecular changes would only become stronger with a bigger study population. Our objective was to capture true PAD patients undergoing aortic clamping and study the observed mechanisms. These patients were scarce. During the study period several open AAA procedures were performed. These patients were not included, because the baseline values of cytokines and purinergic signaling can be significantly altered due to PAD (Jalkanen et al. 2015). An additional limitation is that we only focused on two distinct time points: the morning prior to the operation and the morning of the first post-operative day. However, there is a clear rationale for this. IL-6 has been shown to be a key measure of SIRS and predict poor outcome in several conditions (Sakon et al. 1996; Matsushima et al. 2004; Nieminen et al. 2014), including major surgery (Yamada et al. 1998; Namekata et al. 2000). IL-6 levels peek at the first post-operative day, at around 24 h, while for example CRP rises later on after a major surgery (Vasdekis et al. 2008; Watt et al. 2015). Similarly, organ failures commonly start to develop during the first post-operative day and become evident at the second, or even on the third post-operative day. Thus, we postulate that the first post-operative day is a watershed and as IL-6 peeks then, the repair mechanisms should also be in action at this time point. From the studies reporting increased CD73 expression in humans (Bellingan et al. 2014) we can also postulate that after HIF-1α activation CD73 expression should peek at the first post-operative day.

## Conclusions

Open infra-renal aorta repair is associated with a systemic inflammatory response, but in addition according to the present study an impaired CD39 and CD73 response to attenuate inflammation. This helps to explain severe morbidity and mortality related to open aorta repair. In conclusion, we wish to point out that despite a small study the witnessed phenomenon is obvious and merely proposes an explanatory molecular mechanism behind the burden of aortic clamping. Hypoxia induced inflammation and impaired hypoxia related repair mechanisms seen here relating to open aorta repair require further research and could serve as a target for operative preconditioning and better patient outcome. Such a therapy could be pre-operative statin loading, which is in common use for example in coronary artery bypass grafting (CABG). Statins are known for their anti-inflammatory properties and have been shown to have beneficial effects on CD39 and CD73 levels (Kaneider et al. 2002; Jalkanen et al. 2015). Pre-operative statin therapy has also been associated with better outcome after major vascular surgery (Durazzo et al. 2004; Le Manach et al. 2011; Sanders et al. 2013). However, we still lack a decisive multicenter randomized trial on the effect statin loading on infra-renal aorta repair against SIRS and organ dysfunction. Another possible therapy could be pre-operative administration of intravenous interferon beta. It is known to up-regulate CD73, attenuate inflammation and prevent vascular leakage especially in the lungs (Kiss et al. 2007), and has shown promising initial results in the treatment of ARDS in humans (Bellingan et al. 2014).

## Methods

### Study subjects

As a part of the PURE ASO Study (*The Role of Purinergic Signaling in Atherosclerosis*, approved by the local Ethical Committee of the Hospital District of Southwestern Finland) consecutive blood samples were gathered for 1 year from non-urgent elective PAD patients admitted to the Department of Vascular Surgery of Turku University Hospital, Finland (Jalkanen et al. 2015). The department is a primary vascular surgery referral center for a population of 360,000 inhabitants. During enrollment 227 suitable patients were seen and 226 gave written informed consent. Out of these 226 PAD patients 6 went through open infra-renal abdominal aorta repair (1 for Leriche’s Syndrome, and 5 for TASC D aorta-iliac lesions accompanied with a repairable AAA). For these 6 subjects undergoing aortic clamping 6 age and risk factor matched subjects undergoing common femoral artery clamping during the same hospital stay as the aortic reconstructions were randomly picked as control subjects (5 of these subjects had femoral endarcterectomy done and 1 subject underwent a femoropopliteal bypass to the proximal popliteal artery with a vascular prostheses). All 12 study subjects had claudication as their manifestation of PAD. For these 12 study subjects both pre- and post-operative blood samples were drawn. Otherwise, the PURE ASO Study population only went through the pre-operative blood sampling.

### Blood sampling and analyses

All samples were serum from centrifuged whole blood samples and plasma EDTA. The first samples were drawn in the morning of the operative day after over night fasting. The second sample was drawn the following morning after the operation. At this point patients were still fasting. After centrifugation the serum samples were stored at −70 °C until analyses. All analyses were done at once with the same magnetic bead suspension array kit of Bio-Plex Pro Human Cytokine 21- and 27-plex panels (Bio-Rad, Hercules, CA, USA) according to the manufacturer’s instructions except that the amount of beads, detection antibodies and streptavidin–phycoerythrin conjugate were used at half of their recommended concentration as described previously (Nieminen et al. 2014). The results were analyzed using the Bio-Plex 200 System, and calculated using the Bio-Plex Manager 6.0 software (Bio-Rad Laboratories, Hercules, CA, USA). The persons doing the cytokine analysis were unaware of patient procedures.

The 21-plex panel included interleukin 1α (IL-1α), IL-2 receptor α (IL-2Rα), IL-3, IL-12p40, IL-16, IL-18, cutaneous T cell attracting chemokine (CTACK), growth-regulated oncogene α (GROα), hepatocyte growth factor (HGF), interferon α2 (IFN-α2), leukemia inhibitory factor (LIF), monocyte chemotactic protein 3 (MCP-3), macrophage colony-stimulating factor (M-CSF), macrophage migration inhibitory factor (MIG), monokine induced by IFN-γ (MIF), β-nerve growth factor (β-NGF), stem cell factor (SCF), stem cell growth factor β (SCGF-β), stromal cell–derived factor 1α (SDF-1α), tumor necrosis factor β (TNF-β) and TNF-related apoptosis inducing ligand (TRAIL). The 27-plex included IL-1β, IL-1 receptor antagonist, IL-2, IL-4, IL-5, IL-6, IL-7, IL-8, IL-9, IL-10, IL-12p70, IL- 13, IL-15, IL-17, basic fibroblast growth factor (bFGF), eotaxin, granulocyte colony-stimulating factor (G-CSF), granulocyte- macrophage colony-stimulating factor (GM-CSF), IFN-γ, IFN-γ-induced protein 10, monocyte chemotactic protein 1 (MCP-1), macrophage inflammatory protein 1α (MIP-1α), MIP-1β, platelet-derived growth factor (PDGF), regulated on activation normal T cell expressed and secreted (RANTES), TNF-α and vascular endothelial growth factor (VEGF).

#### Measurement of soluble nucleotidase (CD39 and CD73) activities in human serum

For ADPase/NTPDase activity, serum (10 μL) was incubated for 60 min at 37 °C in 80 μL of RPMI-1640 medium containing 5 mM β-glycerophosphate, 80 μM adenylate kinase inhibitor Ap_5_A, and 50 μM ADP with a [2,8-^3^H]ADP tracer (Perkin Elmer, Boston, USA). Likewise, 5′-nucleotidase activity was assayed by incubating 10 μL of serum for 60 min with 300 μM [2-^3^H]AMP (Quotient Bioresearch, GE Healthcare, Rushden, UK). Radiolabelled substrates and their dephosphorylated products were separated by thin-layer chromatography and quantified by scintillation β-counting. Enzymatic activities were expressed as nanomoles of ^3^H-substrate metabolized per hr by 1 mL of serum (Yegutkin 2015).

#### Quantification of ATP and ADP levels in human plasma

Briefly as described previously (Yegutkin et al. 2003), 10 µL aliquots of EDTA plasma were transferred into two parallel wells of a white non-phosphorescent 96-well microplate containing 100 µL of PBS with (A) or without (B) a solution of 200 µM UTP and 5 U/mL NDP kinase from baker’s yeast *S. cerevisiae* (Sigma). Following the addition of 50 µL of ATP-monitoring reagent, sample luminescence was measured using a Tecan Infinite M200 microplate reader (Salzburg, Austria). The difference in luminescence signals between well “A” (ATP + ADP) and “B” (only ATP) enabled the quantification of ADP concentration, which was converted into ATP through an NDP kinase-mediated reaction in the presence of exogenous UTP. This approach allows simultaneous measurement of both ATP and ADP content within the same sample.

#### Measurement of HIF-1a Activity in Human serum

HIF-1a activity of serum samples was analyzed using ELISA kit of Elabscience (Wuhan, China) according to the manufacturer’s instructions. The optical density (OD) values were read using Tecan Infinite M200 and Magellan 7.2 software for Microsoft Windows (Tecan Group, Männedorf, Switzerland).

### Statistical analysis

Statistical analyses were performed using JMP 11.1 Pro statistical software from SAS (SAS Institute Inc., Cary, NC, USA). Baseline characteristics of subjects are reported using medians and inter-quartile range (IQR). Comorbidities were gathered on a yes/no basis and are presented as a percentage of prevalence amongst subjects. Difference between prevalence was compared using the Chi square test, and difference between numeric values between the groups using the Mann–Whitney U test. Cytokine results are given as median and IQR. Changes in cytokine levels after clamping are presented as percentage rise or fall from the baseline value. The significance of the change of each cytokine level and component of purinergic signaling in relation to clamping was measured using matched pair values across time and the Wilcoxon signed rank test. Cytokine levels that changed significantly because of clamping were then compared between the two study groups (aorta vs. common femoral artery) using the Mann–Whitney U test.

## Additional file

10.1186/s40064-015-1651-x Raw data on patient baseline characteristics, procedural variables, and essential pre- and post-operative analyses.
